# Machine Learning and Experimental Verification Identify Anti-Influenza Natural Products

**DOI:** 10.3390/ijms27125399

**Published:** 2026-06-15

**Authors:** Feifan Qiu, Jiajing Wu, Yan Cao, Xuena Li, Shuo Wang, Kun Xue, Yueqi Wang, Yizhou Bu, Beilei Shen, Yuwei Gao

**Affiliations:** 1 College of Integrated Chinese and Western Medicine, Changchun University of Chinese Medicine, Changchun 130117, China; 2 State Key Laboratory of Pathogen and Biosecurity, Key Laboratory of Jilin Province for Zoonosis Prevention and Control, Changchun Veterinary Research Institute, Chinese Academy of Agricultural Sciences, Changchun 130122, China

**Keywords:** influenza A virus, calmodulin, sodium deoxycholate, deoxycholic acid, machine learning, pulmonary injury

## Abstract

The influenza A virus (IAV) has been responsible for multiple seasonal epidemics and poses a pandemic threat, and the growing number of variant strains constitutes a persistent threat to humanity. This study aimed to identify anti-influenza compounds from a traditional Chinese medicine (TCM) monomer library using a machine learning approach, with calmodulin as a hypothesis-driven target. The antiviral efficacy of the compounds with the highest predicted binding scores from virtual screening was evaluated using integrated computational and experimental approaches. A pre-trained protein language model (ConPLex) was employed for virtual screening. Molecular docking was used to predict binding characteristics, and network pharmacology was applied to generate hypotheses on multi-target mechanisms. The cytotoxicity and anti-H1N1 activity of the selected compounds were assessed in vitro, followed by in vivo evaluation of survival, lung pathology, viral load, and inflammatory mediators in a lethal mouse infection model. Sodium deoxycholate (NaDC) and deoxycholic acid (DCA) were identified as promising lead compounds. Both exhibited dose-dependent inhibition of viral replication in vitro with low cytotoxicity. Treatment with NaDC and DCA significantly improved survival rates and reduced lung pathology in H1N1-infected mice. Treatment was associated with suppression of nuclear factor kappa-B (NF-κB) activation, reduced pro-inflammatory cytokines, and elevated interleukin-10 (IL-10) levels. Molecular docking predictions indicated that NaDC and DCA exhibit moderate binding affinity for calmodulin, with binding energies of −8.38 kcal/mol and −7.61 kcal/mol, respectively. Furthermore, network pharmacology analysis suggested that these compounds may modulate pathways related to viral infection, inflammation, and immune regulation. NaDC and DCA demonstrate anti-influenza activity both in vitro and in vivo, reducing viral replication and alleviating inflammatory lung injury. These findings position NaDC and DCA as promising lead compounds for anti-influenza drug development and provide a foundation for further mechanistic validation.

## 1. Introduction

As the primary causative agent of influenza, influenza A virus (IAV) has triggered four global pandemics, exerting a severe impact on human society [1]. With continuous improvements in research methodologies and data sources, recent studies suggest that previous estimates of the global burden of influenza may have been underestimated. A 2018 study coordinated by the World Health Organization (WHO) and the U.S. Centers for Disease Control and Prevention (CDC) reported that influenza-associated deaths attributable to respiratory diseases alone ranged from 290,000 to 650,000 annually [2], a range subsequently adopted by the WHO as the official estimate. Furthermore, the 2019 Global Burden of Disease (GBD) study estimated that the annual number of lower respiratory tract infection deaths directly attributable to influenza virus infection ranged from 99,000 to 200,000 [3]. Post-infection, patients typically present with localized respiratory symptoms like fever, cough, and pharyngalgia, and are often accompanied by significant systemic inflammatory reactions such as headache and widespread muscle aches [4]. Due to the rapid progression and swift transmission of influenza A infection, some cases can evolve into critical complications like severe pneumonia and acute respiratory distress syndrome (ARDS) [5], potentially resulting in death. This presents a persistent challenge that researchers must confront in future efforts to predict viral evolution and develop vaccines. The use of antiviral drugs represents a crucial intervention for reducing influenza-related morbidity and mortality, playing a particularly vital role in high-risk populations, including immunocompromised individuals, the elderly, and those with underlying medical conditions.

At present, two types of drugs are mainly used in clinical anti-influenza treatment. Among them, neuraminidase inhibitors such as oseltamivir [6] and zanamivir [7] play a role by blocking virus release, while RNA polymerase inhibitors such as baloxavir play a therapeutic role by inhibiting the replication process of the viral genomes. These two classes form the cornerstone of modern influenza pharmacotherapy. IAV undergoes continuous genetic variation through two mechanisms: antigenic drift [8] and antigenic shift [9], leading to the emergence of variant strains with novel combinations of hemagglutinin (HA) and neuraminidase (NA) [10]. However, clinical adverse effects and the continuous emergence of drug-resistant viral strains limit their long-term efficacy [11,12,13]. Therefore, it is urgent to develop new anti-influenza virus drugs that act on new targets or have new mechanisms.

Beyond directly targeting the virus, recent years have seen researchers working to develop therapeutic strategies that target host factors rather than viral components [14,15]. Such strategies may offer advantages in reducing the risk of drug resistance, although their broad-spectrum potential remains to be demonstrated. As a crucial intracellular calcium signal transducer, calmodulin has been documented to participate in the replication cycles of various viruses [16,17]. Following influenza virus infection, intracellular calcium concentrations rise, activating calmodulin and triggering downstream signaling cascades. Calmodulin activation plays a critical regulatory role in both the viral replication cycle and infection-associated tissue damage. Regarding viral replication, calmodulin-dependent kinase (CaMK) promotes the nuclear import of viral ribonucleoprotein complexes by phosphorylating the viral nucleoprotein (vNP) and nuclear pore complexes [18]. In terms of host immune responses, calmodulin activation induces the expression of type I interferons (IFN-α/β) and pro-inflammatory cytokines, representing a core host defense mechanism against viral spread [19]. However, sustained calcium signaling and excessive calmodulin activation can lead to overactivation of the nuclear factor kappa-B (NF-κB) pathway, resulting in a cytokine storm (CS) and contributing to severe influenza pathology [20,21,22,23]. Given the dual regulatory role of the calmodulin signaling pathway in viral replication and inflammatory injury, calmodulin represents a promising host target for the development of anti-influenza drugs.

Traditional Chinese Medicine (TCM) has accumulated systematic theoretical knowledge and extensive practical experience in the prevention and treatment of epidemic diseases. Multi-component TCM formulations with anti-influenza activity, along with the library of TCM-derived monomeric compounds constructed from them for further development, represent a valuable drug resource with significant potential for exploitation. Applying modern computational biology and artificial intelligence technologies for its systematic exploration is a crucial strategy to accelerate the modernization of TCM and the discovery of innovative drugs [24,25].

Our previous study found that the calmodulin antagonist W-7 hydrochloride (W-7) exhibits inhibitory effects against multiple viruses [26,27,28,29] and can effectively alleviate pulmonary inflammatory pathology caused by influenza virus infection by suppressing the activation of the NF-κB pathway. Therefore, using calmodulin as a computational screening anchor, we conducted a large-scale systematic virtual screening of a TCM compound database employing advanced machine learning algorithms. Through in vitro experimental validation, we successfully identified two TCM monomeric compounds, Sodium deoxycholate (NaDC) and deoxycholic acid (DCA), which effectively inhibit the replication of the influenza A H1N1 2009 virus (A/Vinig/01/2009 (H1N1)) (H1N1-UI182) strain in Madin-Darby canine kidney (MDCK) cells at the cellular level.

To further investigate, we established a lethal H1N1-UI182 virus infection model in BALB/c mice to evaluate the in vivo protective efficacy of NaDC and DCA. Given that calmodulin acts upstream of the NF-κB signaling pathway, we examined whether NaDC and DCA modulate this pathway. IAV infection persistently activates NF-κB within host cells [30]. This activation drives the excessive production and release of various cytokines and other inflammatory mediators [31], ultimately contributing to the development of pulmonary inflammatory injury. The balance between pro-inflammatory and anti-inflammatory cytokines is crucial for maintaining immune homeostasis [32]. The transcription factor NF-κB directly binds to the promoter regions of genes encoding pro-inflammatory cytokines, including interleukin-6 (IL-6), interleukin-1β (IL-1β), and tumor necrosis factor-α (TNF-α). This binding potently upregulates their transcription, which serves to amplify the inflammatory cascade [33]. Thus, we analyzed the antiviral and anti-inflammatory properties of NaDC and DCA in relation to their effects on NF-κB activation and cytokine production.

Furthermore, to complement the machine learning-based screening and to computationally explore potential interactions, we performed molecular docking simulations to predict the binding modes between the identified compounds and calmodulin—the target of the known calmodulin inhibitor W-7. Subsequently, network pharmacology analysis was conducted to predict the potential multi-target and multi-pathway mechanisms underlying their anti-influenza effects, thereby constructing a holistic framework from computational prediction to experimental investigation. This study establishes a reusable technical pathway by integrating machine learning-driven virtual screening, in vivo and in vitro efficacy validation, and lead compound discovery, providing a methodological reference for the modernization of Traditional Chinese Medicine research.

## 2. Results

### 2.1. Machine Learning-Based Drug Screening

Employing the drug–target interaction prediction model ConPLex, we screened compounds in the TCM Monomer Compound Library HY-L065 with calmodulin as a hypothetical target. The 2754 small molecules in the database were scored and ranked in descending order based on the predictive scores from the machine learning algorithm (Table S1). Subsequently, based on the virtual screening results, eight TCM monomeric compounds were selected for further experiments using a predicted binding score >0.45 as the selection threshold, combined with considerations of compound accessibility, solubility, and other physicochemical properties (Table 1). These compounds were NaDC, DCA, daucosterol, taurodeoxycholic acid, lathosterol, brassinolide, epibrassinolide, and 5β-cholanic acid.

### 2.2. The Inhibitory Effect of the Compounds on the Virus In Vitro

**Figure 1:**
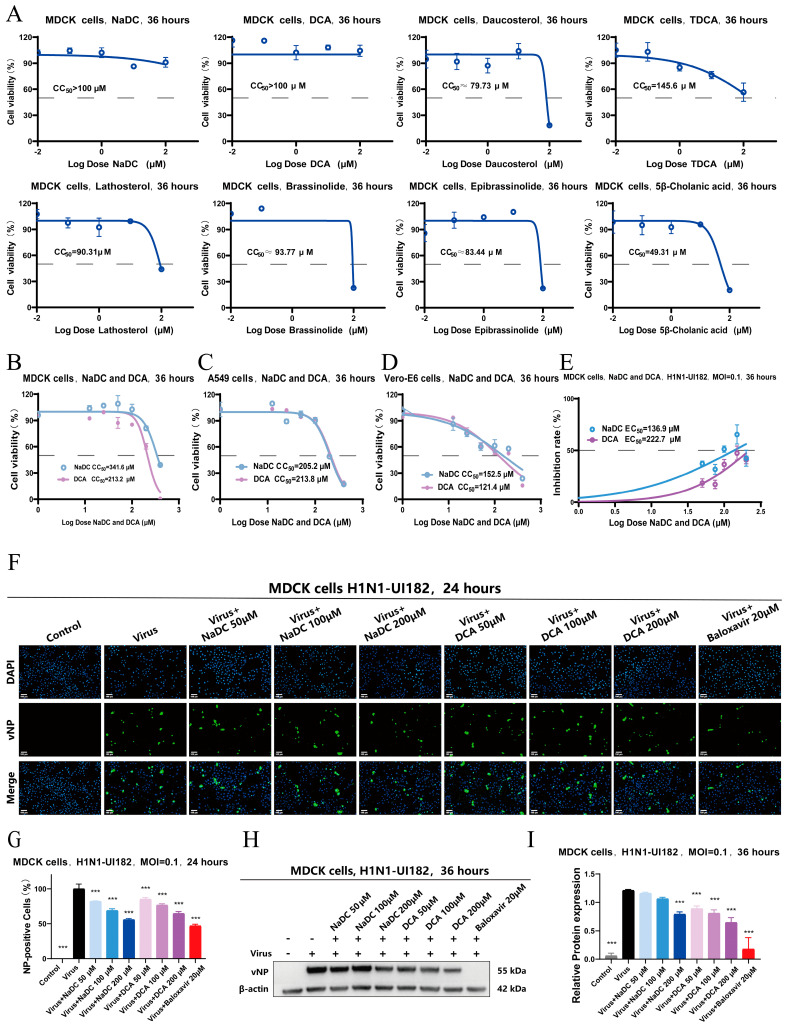
In vitro antiviral evaluation of TCM monomeric compounds against the H1N1-UI182 virus. (**A**) Cytotoxicity screening of eight compounds in MDCK cells. Cell viability was assessed using the CCK-8 assay after 36 h incubation. Data are presented as percentages relative to untreated control cells. At the highest concentration (100 μM), all compounds except NaDC and DCA reduced cell viability compared to control (one-way ANOVA with Dunnett’s test, *p* < 0.05 for each compound except NaDC/DCA). No significant cytotoxicity was observed for any compound at concentrations ≤10 μM (*p* > 0.05). The gray dashed line marks y = 50%. (**B**) MDCK cells were treated with increasing concentrations of NaDC and DCA for 36 h, and viability was measured by CCK-8 assay. One-way ANOVA showed no significant effect on cell viability up to 200 μM for both compounds (*p* > 0.05). (**C**,**D**) Cytotoxicity of NaDC and DCA in A549 cells (**C**) and Vero-E6 cells (**D**), as determined by CCK-8 assay. Both compounds exhibited low cytotoxicity across the tested concentration range (*p* > 0.05 at ≤200 μM, one-way ANOVA). (**E**) Antiviral activity of NaDC and DCA against H1N1-UI182 virus in MDCK cells. Viral inhibition was evaluated by CCK-8 assay, which reflects the protection of cell viability. For each compound, one-way ANOVA with Dunnett’s post hoc test was performed separately. Both NaDC and DCA significantly inhibited viral cytopathic effect at 100 μM and 200 μM compared to the virus control (*p* < 0.001). No significant inhibition was observed at 50 μM (*p* > 0.05). (**F**) Immunofluorescence staining of vNP. MDCK cells were infected with H1N1-UI182 at a multiplicity of infection (MOI) of 0.1 and treated with the indicated concentrations of NaDC or DCA for 36 h. Cells were then fixed and stained with an anti-NP antibody (green). Nuclei were stained with DAPI, and infected cells were detected by NP-specific immunofluorescence staining (scale bar: 100 µm). (**G**) Quantification of NP-positive cells from immunofluorescence images in (**F**). The percentage of NP-positive cells was calculated from at least three random fields per condition. Data are presented as mean ± SD from three independent experiments. *** *p* < 0.001. (**H**) Western blot analysis of vNP expression. MDCK cells were infected with H1N1-UI182 (MOI = 0.1) and treated with the indicated concentrations of NaDC or DCA for 36 h. β-actin was used as the loading control. (**I**) Quantitative analysis of (**H**) was performed using ImageJ software (version 1.54p) (NIH, Bethesda, MD, USA) and GraphPad Prism 8.0.2 software. Data are presented as mean ± SD from three independent experiments. *** *p* < 0.001.

The cytotoxicity of 8 Chinese medicine monomer compounds was first assessed in vitro on MDCK cells using the cell counting Kit-8 (CCK-8) assay. MDCK cells were treated with five different concentrations (0.01, 0.1, 1, 10, 100 μM) of each compound for 36 h. Cell viability assessment revealed that most compounds did not exhibit significant cytotoxicity at concentrations below 10 μM. However, at the highest tested concentration of 100 μM, cell survival was markedly reduced for most compounds (Figure 1A). In particular, NaDC and DCA showed minimal impact on cell viability even at this high concentration. Based on their low cytotoxicity profile, NaDC and DCA were selected for further determination of their half-maximal cytotoxic concentration (CC_50_) in MDCK cells. Subsequent dose–response experiments demonstrated that the CC_50_ values for both NaDC and DCA exceeded 200 μM (Figure 1B).

To determine whether the favorable cytotoxicity profile of NaDC and DCA represents a unique property of these two compounds or a class effect of bile acids, we compared their cytotoxic effects with those of other structurally related steroidal compounds identified during the screening phase. At a concentration of 100 μM, MDCK cells treated with NaDC and DCA exhibited the highest viability (>85%), whereas cell viability following treatment with other steroidal compounds, such as daucosterol and lathosterol, was below 60%. These differences suggest that the dihydroxy substitution pattern on the steroidal skeleton may be associated with low cytotoxicity. Systematic structure–activity relationship (SAR) or structure–toxicity relationship (STR) studies have not yet been conducted, and the specific contribution of each functional group to the observed differences in safety profiles warrants further elucidation through systematic investigations.

To further validate the low cytotoxicity of NaDC and DCA in additional cell lines, cytotoxicity assays in Human Non-Small Cell Lung Cancer (A549) and African Green Monkey Kidney (Vero-E6) cells were performed, which also demonstrated low cytotoxicity for both compounds (Figure 1C,D). Given that both NaDC and DCA were predicted to bind to calmodulin and demonstrated low cytotoxicity, these two compounds were selected for subsequent antiviral experimental validation.

The antiviral activity of NaDC and DCA was further assessed at three different concentrations (50, 100, and 200 μM) in an in vitro model of H1N1-UI182 infection using MDCK cells. Both compounds demonstrated dose-dependent inhibition of viral replication across the tested concentration range, as measured by the CCK-8 assay (Figure 1E). The CCK-8 assay reflects preservation of cell viability as an indirect indicator of reduced viral cytopathic effect. To confirm the antiviral activity, we further assessed vNP expression by immunofluorescence (IFA) and Western blot (WB).

The expression and localization of vNP were assessed by IFA. Quantification of vNP-positive (green fluorescent) cells revealed a concentration-dependent decrease in their number following treatment with either NaDC or DCA, compared to the virus group. At the highest tested concentration (200 μM), both NaDC and DCA caused a marked reduction in vNP immunofluorescence signal intensity, further corroborating the potent inhibitory effect (Figure 1F,G). Furthermore, WB results showed that vNP was expressed at high levels in H1N1-UI182-infected MDCK cells in the virus group. Treatment with either NaDC or DCA led to a concentration-dependent reduction in vNP levels compared to the virus control, demonstrating a dose-responsive inhibition of viral replication by these compounds (Figure 1H,I). In IFA and WB experiments, 20 μM baloxavir was used as a positive control. The results showed that baloxavir treatment almost completely inhibited vNP expression, thereby validating the reliability and effectiveness of the experimental system. These findings laid the foundation for subsequent in vivo testing.

### 2.3. Evaluation of NaDC and DCA in Mice Infected with Lethal H1N1 Virus

**Figure 2:**
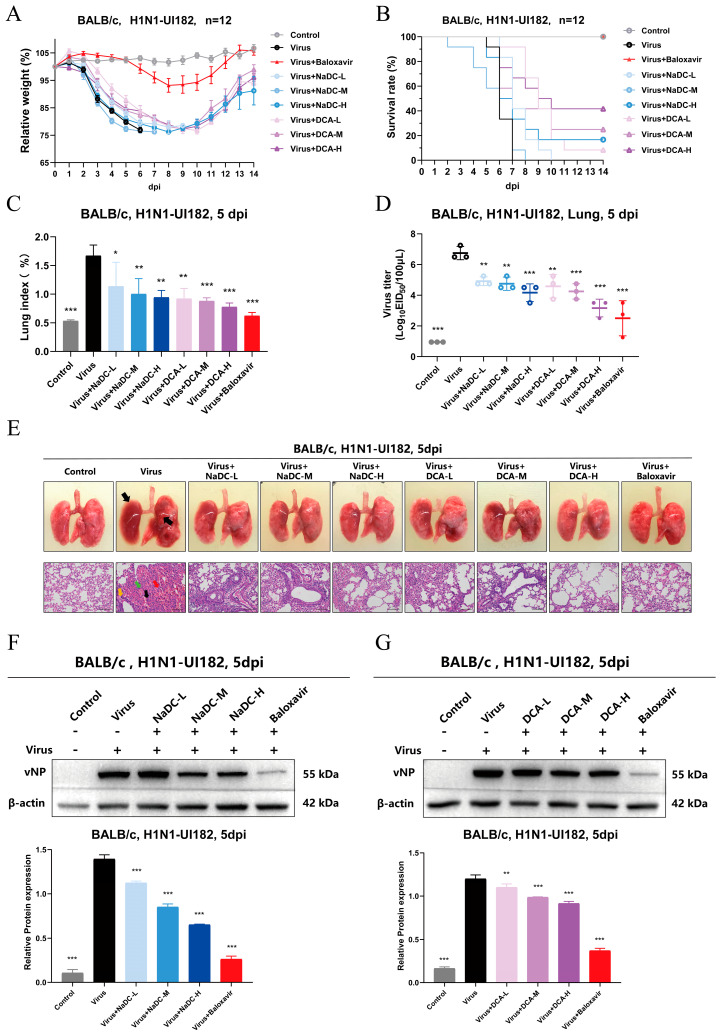
Therapeutic effects of NaDC and DCA in H1N1-UI182-infected mice. (**A**) Body weight changes in control group, virus group (PBS, oral administration), NaDC-L treatment group (25 mg/kg, oral administration), NaDC-M treatment group (50 mg/kg), NaDC-H (100 mg/kg), DCA-L (25 mg/kg), DCA-M (50 mg/kg), DCA-H (100 mg/kg), and baloxavir group (5 mg/kg, subcutaneous injection). All drugs except baloxavir were administered orally. (**B**–**E**) Survival curves for the groups described above (**B**), lung index measured at 5 dpi (**C**), viral titers in lung homogenates at 5 dpi (**D**), and representative lung histology (**E**), with gross appearance (**top**) and H&E-stained sections (**bottom**; scale bar = 200 μm). H&E staining diagram arrow indicates: Black arrow: thickened alveolar wall; green arrow: cellular debris and inflammatory cells in bronchial lumen; red arrow: inflammatory cell infiltration within alveolar wall; orange arrow: red blood cells in alveolar space. Data are presented as mean ± SD (*n* = 3 mice per group from the dissection cohort) and were compared with the virus group. * *p* < 0.05, ** *p* < 0.01, *** *p* < 0.001. (**F**,**G**) WB analysis of vNP expression in lung tissues from infected mice treated with (**F**) NaDC or (**G**) DCA, compared to the virus group. β-actin was used as the loading control. *n* = 3; ** *p* < 0.01, *** *p* < 0.001.

This study employed a lethal mouse model infected with H1N1-UI182 to evaluate the in vivo antiviral efficacy of NaDC and DCA against IAV. Both compounds were orally administered once daily for five consecutive days, starting at 12 h post-infection (hpi), at three dose levels: low (25 mg/kg), medium (50 mg/kg), and high (100 mg/kg). During the experimental period, all mice in the control group survived and showed no signs of clinical illness. In stark contrast, mice in the virus group exhibited a sharp decline in body weight starting on 3 days post-infection (dpi), alongside clinical signs including hunched posture and hypophagia, culminating in 100% mortality by 7 dpi. As expected, treatment with the baloxavir group (5 mg/kg, subcutaneous injection) resulted in 100% survival with minimal body weight loss. Compared with the virus group, all dose groups of NaDC and DCA significantly prolonged the survival time of infected mice, and clinical signs such as fur condition and behavioral activity showed significant improvement. Notably, mice treated with all dose groups of DCA and the high-dose group of NaDC (NaDC-H) exhibited varying degrees of survival protection (Figure 2A,B). The survival rate of the high-dose NaDC group was 16.7%, while that of the high-dose DCA group reached 41.7%.

On 5 dpi, the lung index was significantly elevated in the virus group compared to the uninfected controls. In contrast, treatment with either NaDC or DCA resulted in a dose-dependent reduction in lung index (Figure 2C). Hemagglutination (HA) assay of lung homogenates revealed that both NaDC and DCA treatment caused a dose-dependent reduction in viral titers. The high-dose groups (100 mg/kg) of both compounds reduced lung viral loads by 2–3 log_10_ compared to the virus group (Figure 2D). Collectively, these data demonstrate that NaDC and DCA effectively reduce viral replication and load in the lungs of H1N1-UI182-infected mice.

Gross examination of lungs harvested on 5 dpi revealed severe hemorrhage and edema in the virus group (Figure 2E, black arrow). By contrast, all treatment groups exhibited varying degrees of amelioration in lung pathology, with the most pronounced improvement observed in the NaDC-H (100 mg/kg) and DCA-H (100 mg/kg) groups. Histopathological analysis further confirmed that H1N1-UI182 infection induced substantial lung injury. Hematoxylin and eosin (H&E)-stained lung sections from the virus group exhibited extensive pathological changes. These changes consisted of widespread alveolar wall thickening, inflammatory cell infiltration in the alveolar walls and interstitium, focal alveolar hemorrhage, and the accumulation of red blood cells and cellular debris within alveolar spaces. Lung sections from mice treated with NaDC or DCA showed significantly attenuated inflammatory cell infiltration, rare alveolar dilation, reduced hemorrhage, and an overall alleviation of lung tissue damage.

This study further examined the effect of NaDC and DCA on H1N1-UI182 replication at the protein level in vivo using WB. A significant increase in vNP expression was observed in the virus group, confirming robust viral replication in vivo. Treatment with either NaDC or DCA resulted in a significant and dose-dependent reduction in vNP expression levels (Figure 2F,G).

### 2.4. Investigation of the Molecular Mechanisms of NaDC and DCA

Influenza virus infection induces intracellular calcium homeostasis imbalance [34], which activates calmodulin. As a signal integrator and amplifier during infection, calmodulin promotes activation of the NF-κB signaling pathway through CaMK and other downstream effectors [35,36]. Activated NF-κB directly upregulates the transcription of pro-inflammatory cytokines such as TNF-α, IL-1β, and IL-6, and engages in complex cross-regulation with the type I interferon response [37]. Excessive activation of the NF-κB pathway leads to overproduction of these pro-inflammatory mediators, triggering a CS [38]. In addition to its role in NF-κB activation, calmodulin may further amplify the CS by mediating crosstalk between calcium signaling [39] and other inflammatory pathways.

The pathogenesis of influenza virus involves both virus-induced cytopathic effects and excessive host inflammatory responses [40]. To investigate the intrinsic link between the anti-H1N1 activity of NaDC and DCA and their anti-inflammatory effects via NF-κB pathway inhibition, we selected the two high-dose treatment groups (which showed the highest survival rates in the animal model) for in-depth analysis of lung tissue.

WB analysis of lung tissue revealed that, compared to the control group, the virus group exhibited significantly increased levels of p-p65 and the NF-κB p105/p50 subunit, along with decreased IκBα expression. Consequently, the ratios of p-p65 to total p65 and p-IκBα to total IκBα were markedly elevated in infected mice. Treatment with NaDC-H significantly reduced these elevated protein ratios compared to the virus group. Similarly, the DCA-H group showed significant reductions in p105/p50 protein levels and the p-p65/p65 ratio. The significant downregulation of key protein expression levels in the NF-κB pathway suggests that NaDC and DCA may exert their protective effects against H1N1-UI182 infection in mice, at least in part, by inhibiting the phosphorylation and activation of this pathway.

**Figure 3:**
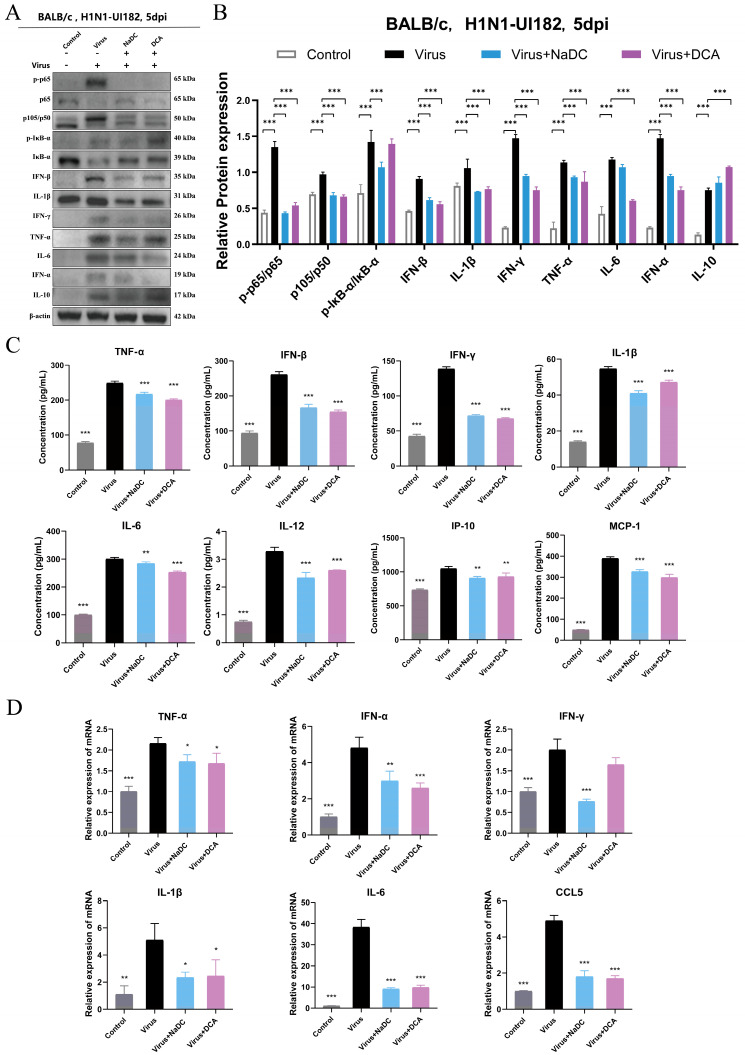
NaDC and DCA attenuate the inflammatory response in the lungs of H1N1-UI182-infected mice. (**A**,**B**) WB analysis of key signaling proteins in the NF-κB pathway (e.g., p-IκBα, p-p65) and downstream pro-inflammatory cytokines (e.g., IL-6, TNF-α) in lung tissues from different treatment groups at 5 dpi. β-actin was used as the loading control. (**C**) Concentrations of pro-inflammatory cytokines (e.g., IL-1β, IL-6, TNF-α) and chemokines (e.g., IP-10, MCP-1) in lung homogenates were measured by ELISA. (**D**) Relative mRNA expression levels of inflammatory cytokines (e.g., IL-6, TNF-α, IL-1β) in lung tissues were determined by RT-qPCR. *β-actin* was used as the loading control. Data are presented as mean ± SD (*n* = 3 mice per group from the dissection cohort) and were compared with the virus group. * *p* < 0.05, ** *p* < 0.01, *** *p* < 0.001.

Concomitantly, H1N1-UI182 infection triggered a substantial increase in pulmonary levels of multiple cytokines and interferons, including IL-1β, IL-6, TNF-α, IFN-α, IFN-β, and interferon-γ (IFN-γ), as well as the anti-inflammatory cytokine interleukin-10 (IL-10). Importantly, treatment with NaDC-H and DCA-H not only further elevated IL-10 expression but also potently suppressed the induction of key pro-inflammatory cytokines (Figure 3A,B).

Enzyme-linked immunosorbent assay (ELISA) analysis of lung homogenates confirmed that H1N1-UI182 infection significantly elevated a broad spectrum of cytokines and chemokines, including TNF-α, IFN-β, IFN-γ, IL-1β, IL-6, interleukin-12 (IL-12), interferon gamma-induced protein 10 (IP-10), and monocyte chemoattractant protein-1 (MCP-1). High-dose treatment with either NaDC or DCA markedly attenuated this infection-induced increase in inflammatory mediators. This suppression of the CS suggests that NaDC and DCA effectively mitigate excessive inflammation and associated tissue injury triggered by H1N1-UI182 infection (Figure 3C).

To further investigate the anti-inflammatory effects at the transcriptional level, we quantified the mRNA expression of multiple pro-inflammatory mediators in mouse lung tissues by real-time quantitative PCR (RT-qPCR). The results demonstrated that treatment with NaDC or DCA significantly downregulated pulmonary mRNA expression of *TNF-α*, *IFN-α*, *IFN-γ*, *IL-1β*, *IL-6*, and *C-C motif chemokine ligand 5* (*CCL5*) compared to the virus group (Figure 3D).

Since calmodulin plays a regulatory role in both viral replication and the upstream activation of NF-κB, the direct and indirect effects of NaDC and DCA are difficult to distinguish. Viral replication itself can activate innate immune signaling; therefore, the observed attenuation of inflammation may be partially attributed to reduced viral load rather than direct intervention of the compounds on the NF-κB pathway. Experimental results further demonstrated that NaDC and DCA inhibited vNP expression in a dose-dependent manner, accompanied by suppression of NF-κB activation and decreased levels of inflammatory cytokines, suggesting that their anti-inflammatory phenotype is at least in part secondary to the inhibition of viral replication. Given that both compounds are predicted to bind to calmodulin, their protective effects may reflect a combination of putative direct effects and indirect regulation. Regardless of the specific mechanism, the strong correlation among reduced viral load, alleviated inflammation, and improved survival supports the further development of NaDC and DCA.

### 2.5. Molecular Docking Simulation of the Two Compounds with Calmodulin

**Figure 4:**
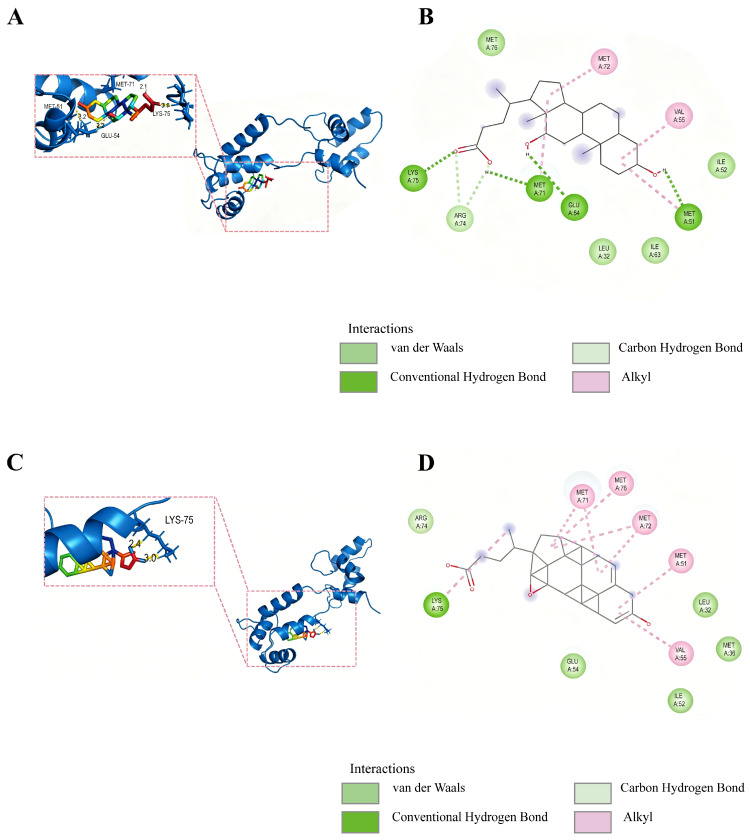
Molecular docking of DCA and NaDC with calmodulin. (**A**,**B**) Docking model and interaction profile of DCA with calmodulin. (**C**,**D**) Docking model and interaction profile of NaDC with calmodulin.

In this study, molecular docking simulations were performed to investigate the interactions between the two compounds and calmodulin, respectively. The docking configurations of both compounds within the calmodulin binding site are illustrated in Figure 4. Analysis of the docking models revealed that DCA formed a hydrogen bond with the residue Lys-75. In contrast, NaDC established multiple hydrogen bonds with residues Met-51, Glu-54, Met-71, and Lys-75. Both compounds also engaged with the calmodulin via van der Waals forces, carbon-hydrogen bonds, and alkyl interactions. The calculated binding free energies for the NaDC and DCA complexes were −8.38 kcal/mol and −7.61 kcal/mol, respectively. These computational predictions suggest possible interactions between the two compounds and calmodulin, warranting experimental investigation. As a positive control, the known calmodulin inhibitor W-7 was docked into calmodulin (PDB ID: 1MUX) using the same protocol. The calculated binding energy of W-7 was −7.63 kcal/mol (Figure S1), which is comparable to those of NaDC (−8.38 kcal/mol) and DCA (−7.61 kcal/mol). The similar binding energies suggest that NaDC and DCA may bind to calmodulin with affinities comparable to that of the established inhibitor W-7, supporting their potential as effective anti-influenza agents targeting calmodulin.

### 2.6. Network Pharmacological Target Prediction 

Network pharmacology analysis was used to predict the potential multi-target, multi-pathway mechanisms underlying the anti-influenza activity of NaDC and DCA. All results are predicted from public databases and have not been experimentally validated. They serve only as a hypothesis-generating tool and should not be interpreted as mechanistic evidence. The analysis suggests that both compounds may exert synergistic effects by regulating pathways related to viral infection, inflammation, and immune responses, which aligns with the observed anti-inflammatory and antiviral phenotypes.

#### 2.6.1. Identification of Potential Overlapping Targets

**Figure 5:**
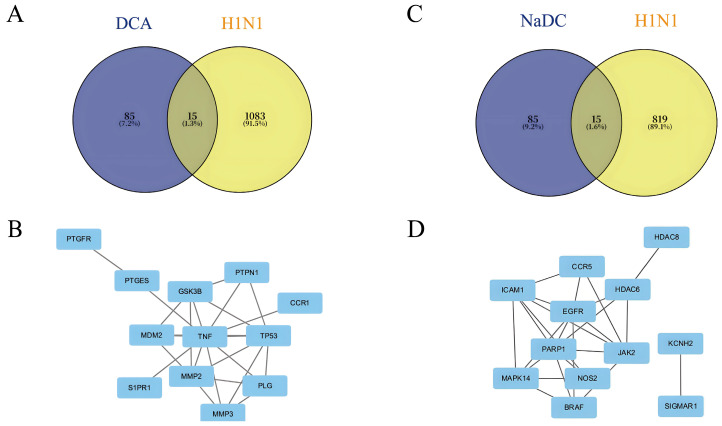
Identification of Potential Targets of DCA and NaDC Against H1N1 Influenza and Analysis of PPI Networks. (**A**,**C**) Venn diagrams showing the intersection between predicted drug targets and H1N1-associated disease targets for DCA (**A**) and NaDC (**C**), respectively. (**B**) PPI network constructed to reveal the interactions among the key common targets between DCA and H1N1 at the protein level. (**D**) PPI network constructed to reveal the interactions among the key common targets between NaDC and H1N1 at the protein level.

Putative targets of DCA and NaDC were retrieved from public databases and intersected with H1N1 influenza-associated disease targets. Venn diagram analysis revealed that both DCA and NaDC shared 15 common targets with H1N1 (Figure 5A,C). These overlapping targets represent candidate nodes through which the compounds might potentially exert their effects.

#### 2.6.2. PPI Network Construction and Core Target Analysis

The 15 common targets for DCA and NaDC were separately used to construct protein–protein interaction (PPI) networks using the STRING database, followed by topological analysis (Figure 5B,D). These targets exhibited high connectivity and occupied hub positions in their respective networks, suggesting they could be functionally important if the predicted interactions are biologically relevant.

#### 2.6.3. KEGG Pathway Enrichment Analysis

**Figure 6:**
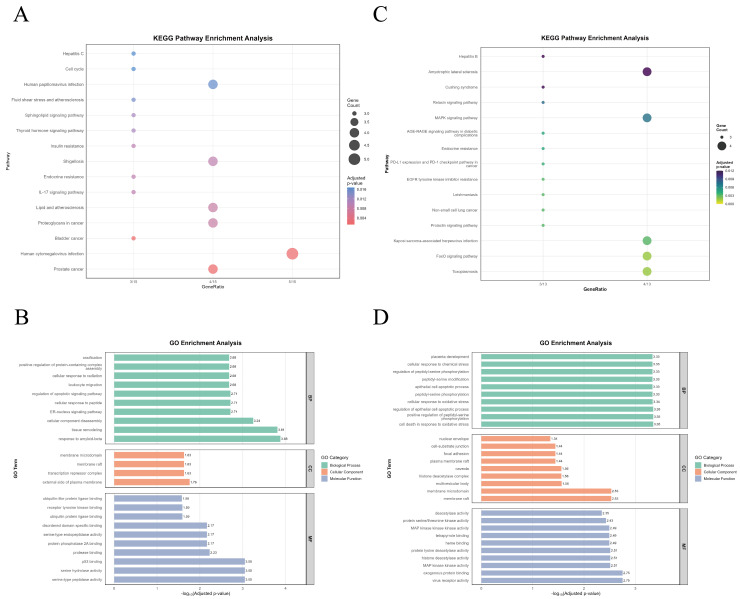
Predicted target enrichment analyses (KEGG and GO) for DCA and NaDC. (**A**) KEGG pathway enrichment analysis for DCA. (**B**) GO functional enrichment analysis of DCA-related targets. (**C**) KEGG pathway enrichment analysis for NaDC. (**D**) GO functional enrichment analysis of NaDC-related targets.

KEGG pathway enrichment analysis was performed on the common targets of each compound (adjusted *p*-value < 0.05). Targets of DCA were significantly enriched in pathways related to viral infection (e.g., Human cytomegalovirus infection, Human papillomavirus infection), inflammation/immunity (e.g., IL-17 signaling pathway), cell cycle, and metabolism (e.g., Insulin resistance) (Figure 6A). Targets of NaDC were significantly enriched in pathways involving viral infection, key signaling transduction (e.g., MAPK signaling pathway, FoxO signaling pathway), and disease/drug resistance (e.g., EGFR tyrosine kinase inhibitor resistance, Endocrine resistance) (Figure 6C). Collectively, these results suggest a complex multi-pathway network for both compounds. These in silico predictions provide a hypothetical framework and suggest specific directions for future experimental validation.

#### 2.6.4. Gene Ontology (GO) Functional Enrichment Analysis

GO analysis further delineated the functional attributes of the targets. For DCA, significant enrichment was observed in molecular functions such as “ubiquitin-like protein ligase binding”, “p53 binding”, and “serine hydrolase activity”, (Figure 6B). For NaDC, significant enrichment was found in cellular components including the “nuclear envelope”, “focal adhesion”, “plasma membrane raft”, and “histone deacetylase complex”, (Figure 6D).

The network pharmacology analysis predicts that both DCA and NaDC may exert anti-H1N1 effects by acting on a set of 15 key targets and synergistically regulating a network encompassing viral infection, immune-inflammatory responses, and cellular signaling. These predictions provide a valuable theoretical framework and specific directions for subsequent experimental validation of their mechanisms and potential synergy.

#### 2.6.5. Experimental Validation of Predicted Signaling Pathways

KEGG pathway enrichment analysis revealed that the potential targets of DCA were enriched in pathways including the IL-17 signaling pathway, whereas those of NaDC were enriched in pathways such as the MAPK signaling pathway. This suggests that the two structurally similar compounds may exert their anti-inflammatory and antiviral effects through partially distinct signaling networks. To assess whether the pathways predicted by network pharmacology are functionally relevant, we examined two representative indicators: IL-17 protein level and p38 MAPK phosphorylation status. Western blot analysis showed that, compared with the virus control group, DCA treatment significantly reduced IL-17A protein levels to nearly those of the control group (Figure S1), whereas NaDC treatment markedly inhibited p38 MAPK phosphorylation without altering total p38 expression (Figure S2B). These results provide preliminary validation of the network pharmacology predictions and suggest that DCA and NaDC may act in a multi-target, multi-pathway manner during influenza infection.

## 3. Discussion

IAV infection continues to pose a significant global health burden, and the emergence of drug-resistant strains underscores the urgent need for antivirals with novel mechanisms of action. Strategies that target host factors, including those hypothetically directed at calmodulin, have attracted increasing interest due to their potential for higher barriers to resistance. In this study, we employed a machine learning-assisted virtual screening approach to identify potential calmodulin-interacting compounds from a TCM monomer library, and identified NaDC and DCA as possessing anti-influenza activity. Our in vitro and in vivo experiments demonstrated that both compounds exhibit dose-dependent antiviral activity against H1N1-UI182 strain and ameliorate virus-induced pulmonary pathology in a lethal mouse infection model.

Given that calmodulin acts as an upstream regulatory node of the NF-κB activation pathway [34,36,41], this study further investigated whether NaDC and DCA exert modulatory effects on this signaling pathway. Infection with H1N1-UI182 activates the NF-κB signaling pathway in mice [42]. Activated NF-κB further induces the expression of the negative regulators IκBα and p105 [43], thereby constituting a classical feedback regulatory loop. WB analysis demonstrated that treatment with either NaDC or DCA significantly reduced the ratios of p-p65 to total p65 and p-IκBα to total IκBα, and also decreased the expression level of the NF-κB subunit p105/p50.

The excessive activation of the NF-κB pathway during the peak of viral replication serves as a critical initiating event for the CS [44]. This excessive activation drives the oversecretion of key pro-inflammatory cytokines, including TNF-α, IL-1β, and IL-6. The elevated cytokine levels increase vascular and alveolar epithelial permeability, resulting in pulmonary hemorrhage and edema [45]. By inhibiting the expression of TNF-α, IL-1β, and IL-6, NaDC and DCA effectively disrupt the inflammatory signaling cascade—an effect that likely reflects the hypothesized ability to interfere with calmodulin-mediated upstream signaling. This action helps protect the integrity of lung tissue, thereby alleviating pathological manifestations such as pulmonary hemorrhage and edema.

Type I interferons are pivotal effector molecules of the antiviral innate immune response [46]. However, during peak viral replication, this coordinated response becomes dysregulated. Overproduction of IFN-α/β not only directly potentiates NF-κB activity but also upregulates the expression of chemokines like CCL5. CCL5 recruits neutrophils, whereas factors like MCP-1 recruit monocytes/macrophages. Activated natural killer (NK) cells produce IFN-γ, which acts as a potent activator of NF-κB. This activation amplifies the immune response and concurrently induces substantial production of IP-10. This complex positive feedback loop ultimately leads to the infiltration and activation of a large number of inflammatory cells and immune cells in the lungs [47].

Consistent with the observed reduction in viral load, treatment with NaDC and DCA was associated with lower levels of IFN-α, IFN-β, and IFN-γ, as well as decreased expression of the chemokines CCL5, MCP-1, and IP-10. These changes likely contribute to reduced inflammatory cell infiltration and lung tissue injury. Since these inflammatory cells themselves are potent sources of further NF-κB activation and cytokine production, this dual inhibitory effect—suppressing the initial signal while blocking cell recruitment—effectively curbs the cascade amplification of inflammation in the lungs. Beyond driving pro-inflammatory responses, the NF-κB pathway can also induce the expression of anti-inflammatory mediators, including IL-10. IL-10 plays a crucial role in dampening excessive inflammation, maintaining immune homeostasis, and facilitating tissue repair [48]. Mechanistically, IL-10 exerts its anti-inflammatory effects by suppressing pro-inflammatory cytokine production in macrophages and dendritic cells. Furthermore, it facilitates tissue repair and accelerates lung recovery through mechanisms that include promoting alveolar epithelial cell proliferation and stimulating angiogenesis.

Collectively, the data from WB, ELISA, and RT-qPCR suggest that NaDC and DCA likely exert their antiviral and immunomodulatory effects by suppressing the NF-κB signaling pathway and the consequent overproduction of downstream inflammatory cytokines [49]. NaDC and DCA not only suppress multiple pro-inflammatory factors but also upregulate the IL-10-mediated endogenous anti-inflammatory pathway, thereby helping to restore immune homeostasis. This allows the function of IL-10 in limiting and terminating immune responses not to be overwhelmed by the massive pro-inflammatory signals, protecting the host from damage caused by excessive immune responses [50]. These findings support the hypothesis that NaDC and DCA can exert dual therapeutic effects—antiviral and anti-inflammatory—a mechanism that closely aligns with the traditional Chinese medicine principle of “supporting vital energy and eliminating pathogens” [51]. Drugs capable of balancing viral replication inhibition and immunopathological modulation hold promise for significantly reducing the risk of disease progression to severe stages and lowering the incidence of post-infection sequelae.

Bile acids, including deoxycholic acid and its sodium salt, have traditionally been recognized primarily for their roles in lipid digestion and as signaling molecules involved in metabolic regulation. In recent years, accumulating evidence has indicated that bile acids also modulate inflammatory responses and related metabolic pathways. This finding aligns theoretically with the “clearing heat and detoxifying” effects [52] attributed to bile-containing traditional Chinese medicines (such as bear bile [53] and bezoar [53]) in the clinical treatment of febrile and infectious diseases. The present study demonstrates that NaDC and DCA, which are primary and secondary components of bile acids, possess anti-influenza activity, further supporting this theoretical framework.

The computational approaches employed in this study provided complementary perspectives on the mechanisms of NaDC and DCA. The rationale for selecting NaDC and DCA lies in their potential to target the host factor calmodulin, thereby regulating viral replication and inflammatory signaling pathways. To test this hypothesis, molecular docking simulations were performed in this study, and the results showed that both NaDC and DCA could bind to calmodulin, with binding energies of −8.38 kcal/mol and −7.61 kcal/mol, respectively. It should be noted that molecular docking is a computational prediction tool, and although the binding energies suggest the possibility of favorable intermolecular interactions, they do not confirm calmodulin-related affinity, binding specificity, or functional modulatory capacity. Therefore, while the docking results are helpful for hypothesis generation, direct target validation using biophysical methods such as surface plasmon resonance (SPR) or isothermal titration calorimetry (ITC) is still required. Network pharmacology analysis suggested that these compounds may regulate a complex network of biological processes, with their predicted targets significantly enriched in pathways related to viral infection, inflammatory/immune responses (e.g., the IL-17 signaling pathway), and key cellular signaling pathways (e.g., the MAPK signaling pathway), indicating that they may exert their pharmacological effects through a multi-target synergistic manner. Although this prediction is consistent with the observed anti-inflammatory phenotype and can provide useful hypotheses for downstream studies, further experimental validation is still required. Nevertheless, although network pharmacology results should not be interpreted as mechanistic evidence, they provide useful hypothesis-generating insights for guiding future experimental studies.

NaDC and DCA should currently be regarded as lead compounds rather than definitive drug candidates. Their low cytotoxicity and oral efficacy in a lethal infection model provide a solid foundation for further lead optimization. Furthermore, the administration route and dosage used in this study cannot be directly extrapolated to the traditional oral use of bile-containing preparations. Future targeted pharmacokinetic studies are needed to fill critical data gaps in translational research.

This study demonstrates the anti-influenza activity of NaDC and DCA, and several questions remain for future investigation. First, the antiviral activity of NaDC and DCA has currently been evaluated only against the H1N1-UI182 strain, and data on other influenza A virus subtypes (e.g., H3N2) as well as influenza B virus lineages are lacking. Future studies should expand the antiviral spectrum to comprehensively assess their clinical translational potential. Second, the calmodulin-targeting hypothesis remains to be experimentally validated. Future studies will employ biophysical methods such as SPR to verify the direct binding of NaDC and DCA to calmodulin. Third, SAR and STR studies, including comparisons with structurally related bile acid derivatives, would help elucidate the structural determinants affecting efficacy and safety. Fourth, pharmacokinetic and oral bioavailability evaluations will be important to further assess their clinical translational potential.

Overall, NaDC and DCA represent promising lead compounds that warrant further development toward therapeutic application. Future studies addressing these questions will help more accurately evaluate their practical value and translational prospects.

## 4. Materials and Methods

### 4.1. Virtual Screening

This study employed ConPLex, a machine learning method for predicting drug–target interactions, to screen small-molecule compounds from TCM targeting the calmodulin. ConPLex was selected for this virtual screening due to its integrated design of pre-trained protein language model and contrastive learning strategy, which overcomes the limitation of traditional sequence models in balancing generalization ability and specificity. Pre-trained protein language model extracts deep protein features through pre-training on large-scale protein sequences to compensate for the scarcity of drug–target interaction (DTI) data, while contrastive learning is trained via triplet loss to cluster real interacting protein-ligand pairs in the embedding space and keep them far from non-interacting decoy compounds with similar physicochemical properties. ConPLex predicts drug–target binding by projecting both protein and compound representations into a shared latent space and calculating the cosine distance between them, which is then converted into an interaction probability via a sigmoid activation function. A shorter cosine distance (i.e., higher predicted binding score) indicates a higher likelihood of binding. This embedding-based prediction mechanism enables rapid, large-scale screening without requiring structural information or pair-wise computation for each new candidate, making it highly suitable for virtual screening of traditional Chinese medicine monomer libraries. In addition, the prediction performance of ConPLex has been experimentally verified by its development team: 12 out of 19 protein-ligand pairs screened in an unbiased manner had a dissociation constant (Kd) of less than 100 nM, among which 4 pairs achieved sub-nanomolar binding affinity, and all were consistent with known interactions reported in the literature. The TCM compounds selected in this study were all the highest-score prediction results of ConPLex, and their confidence is directly supported by the experimentally verified high prediction accuracy of the algorithm. ConPLex also features fast prediction speed, which can maintain a high recall rate in large-scale TCM monomer library screening, further ensuring the reliability of screening results. The compound library was sourced from the MedChemExpress TCM Monomer Compound Collection. The open-source algorithm code was obtained from ConPLex.csail.mit.edu [54]. Key model parameters were configured according to the original publication, including: protein features extracted using the ProtBert pre-trained model (dt = 1024), compound features encoded as Morgan fingerprints (dm = 2048), and final projection into a shared embedding space of dimension h = 1024. The model was trained using the AdamW optimizer with learning rates set to 1 × 10^−4^ (cross-entropy stage) and 1 × 10^−5^ (contrastive learning stage), and all computations were performed on an NVIDIA A100 GPU((NVIDIA Corporation, Santa Clara, USA).

### 4.2. Molecular Docking and Visualization

To elucidate the interaction mechanisms of NaDC and DCA with the target calmodulin, molecular docking simulations were performed. The 3D structures of NaDC and DCA were retrieved from the ZINC20 database (https://zinc20.docking.org/) and prepared using Open Babel. The receptor structure (PDB ID: 1MUX) is the crystal structure of calmodulin. The receptor structure was prepared, and the grid setting for ligand docking was strictly designed following the general norms and standard procedures of the field: based on the original coordinate system of the target protein, the grid center of the potential binding site for ligand docking was defined as X: −16.056, Y: −1.833, Z: −18.278, the grid box size was set to 104 Å × 104 Å × 104 Å (x, y, z) with a grid spacing of 0.375 Å. Molecular docking was performed using AutoDock Vina 1.1.2. The active site of calmodulin (PDB ID: 1MUX) was defined based on the binding region of the known calmodulin inhibitor W-7. Ten docking poses were generated for each compound. The final pose was selected as the one with the lowest binding free energy that also formed plausible interactions with key residues (e.g., Lys-75, Met-51, Glu-54). The Lamarckian genetic algorithm (LGA) was employed for all docking runs. The resulting protein-ligand complexes were visualized and analyzed using PyMOL (version 2.3.0).

### 4.3. Network Pharmacology Analysis

This study employs network pharmacology methods to generate hypotheses regarding the potential multi-target mechanisms of NaDC and DCA. First, the SwissTargetPrediction database (http://www.swisstargetprediction.ch/ accessed on 9 June 2026) was utilized to identify potential protein targets for the identified compounds, with a probability threshold set at >0.1. Predicted targets were standardized to official gene symbols. Subsequently, the human gene database GeneCards was used to collect targets associated with H1N1. The intersection of drug-predicted targets and disease-related targets was taken to obtain potential therapeutic targets. The STRING database (version 11.5) was utilized to construct a PPI network for these shared targets, with a minimum interaction confidence score set at 0.4 (medium confidence). Visualization and analysis of the resulting network were performed using Cytoscape software (version 3.9.1). Its CytoHubba plugin was employed to calculate key topological parameters including degree, betweenness centrality, and closeness centrality, to identify core targets. Finally, functional enrichment analysis of the common targets was conducted using the DAVID bioinformatics database (version 6.8).

### 4.4. Cell Experiments

The mouse-adapted H1N1-UI182 virus strain used in this study was obtained from the Institute of Changchun Veterinary Research, Chinese Academy of Agricultural Sciences (Changchun, China). The A549 cell line, Vero-E6 cell line, and MDCK cell line were obtained from the same institute [55,56,57]. The H1N1 virus was inoculated into the MDCK cell line. All cell lines were cultured in DMEM with 10% fetal bovine serum (FBS, Gibco Lot.No.6125170, Waltham, MA, USA) supplemented with 100 IU/mL Penicillin and 100 μg/mL Streptomycin.

8 TCM monomer compounds (CAS No. 302-95-4; 83-44-3; 474-58-8; 516-50-7; 80-99-9; 72962-43-7; 78821-43-9; 546-18-9) and baloxavir covered in this article were purchased from Shanghai Topscience Co., Ltd. (Shanghai, China).

The viability of MDCK cells was assessed using a CCK-8 assay (Beyotime, Shanghai, China) following the manufacturer’s instructions. MDCK cells (8000 cells/well) were seeded in 96-well plates. The cells were incubated with different concentrations of compounds for 36 h to evaluate their potential cytotoxicity. The absorbance at 450 nm was measured for all groups, including the drug-treated groups and the control group (uninfected group). MDCK cells without drug intervention were used as the control group, and the absorbance was measured at 450 nm with the drug group. Cell viability of A549 cells and Vero-E6 cells was tested in the same manner. The cell viability rate was calculated as follows: (ODexperiment − ODcontrol)/(ODcontrol − ODblank) × 100%.

The 50% tissue culture infectious dose (TCID_50_) was determined by the Reed-Muench method and the infectious virus titer in the cell culture supernatant was determined to further quantify the antiviral effect (Table S2).

For the CCK-8 assay, MDCK cells (8000 cells/well) were seeded in 96-well plates and then exposed to H1N1-UI182 (MOI = 0.1) for 1 h. Following removal of the supernatant, the cells were rinsed with phosphate-buffered saline (PBS) and cultured in medium for 36 h. After incubation with CCK-8 reagent for 1 h at 37 °C, the OD of the cells was measured at 450 nm. The inhibition rate was calculated as follows: (ODexperiment − ODvirus)/(ODcontrol − ODvirus) × 100%.

### 4.5. WB

Cells in culture plates or tissue samples in 1.5 mL tubes were lysed by adding an appropriate volume of ice-cold lysis buffer (P0013B, Beyotime, Shanghai, China) supplemented with protease inhibitors. For cells, lysis proceeded on ice for 30 min. For tissues, lysis beads were added to the tubes, and homogenization was performed using a tissue lyser at 4 °C (30 Hz for 360 s). Following lysis, samples were centrifuged at 14,500× *g* for 15 min at 4 °C. The resulting supernatant containing the solubilized proteins was carefully collected. The protein concentration of each sample was quantified using the BCA assay (23225, Thermo, Shanghai, China). Subsequently, all samples were normalized to a uniform protein concentration. The normalized protein lysates were then mixed with 5× SDS-PAGE loading buffer (P0015L, Beyotime, China) at a 4:1 ratio (sample: buffer) and denatured by heating at 100 °C for 5 min. Equal amounts of protein (10 μg per lane) from each sample were loaded onto a SurePAGE™ precast gel (M00719, Genscript, Nanjing, China). Electrophoresis was then carried out in 1× SDS running buffer (M00680-500, Genscript, China) at a constant voltage of 120 V for 55 min. Following electrophoresis, proteins were transferred from the gel onto an activated PVDF membrane (Thermo, China) using a wet transfer system. The transfer was performed at a constant current of 300 mA for 90 min in a cold room to maintain a temperature of approximately 4 °C. The membrane was then blocked with 5% bovine serum albumin (BSA; Sigma-Aldrich, Beyotime, China) for 1 h at room temperature (RT) on a shaking platform. Following blocking, the membrane was incubated overnight at 4 °C in the appropriate primary antibody solution. Following the primary antibody incubation, the membrane was washed three times with 1× TBST buffer for 5 min each. Subsequently, it was incubated with an appropriate horseradish peroxidase (HRP)-conjugated secondary antibody for 1 h at RT. After washing the membrane three times with 1× TBST, chemiluminescent detection was performed. The membrane was incubated with the pre-mixed ECL substrate solution for 30–60 s in the dark. The protein bands were then visualized and images captured using a chemiluminescence imaging system. Grayscale analysis and quantification were performed using ImageJ software (NIH, Bethesda, MD, USA), with β-actin as the internal reference protein.

The following antibodies were used: NP (Abcam, Shanghai, China, ab104870), β-actin (Abcam, ab6276), IL-10 (CST, Danvers, MA, USA, D13A11), IL-1β (CST, D3U3E), IL-6 (CST, D5W4V), TNF-α (CST, D2D4), IκBα (CST, 44D4), p-IκBα (CST, 14D4), IFN-α (CST, 6B18), IFN-β (CST, D2J1D), IFN-γ (BIOGOT TECHNOLOGY, Nanjing, China, BS3486), p65 (CST, D14E12), p-p65 (CST, 93H1), NF-κB p105/p50 (Beyotime, AF1246), IL-17A (CST, D1X7L), p-p38 MAPK (CST, 28B10), p38 MAPK (CST, D13E1) and goat anti-rabbit IgG (Beyotime, A0208) and goat anti-mouse IgG (Beyotime, A0216).

### 4.6. IFA

Cells were seeded in 12-well plates. At approximately 45% confluence, they were infected with IAV strain H1N1-UI182 (MOI = 0.1). At 36 hpi, the medium was aspirated, and the cells were washed twice with PBS. The cells were fixed with 4% paraformaldehyde (PFA) for 20 min at RT and then permeabilized with 0.5% Triton X-100 (Beyotime, P0096) for 20 min at RT. Subsequently, nonspecific binding sites were blocked by incubating the cells with 2% BSA in PBS for 2 h at RT. The cells were then incubated overnight at 4 °C with a primary antibody against influenza virus nucleoprotein (rabbit monoclonal, Abcam, ab104870, 1:1000) in blocking buffer. After three washes with PBS, the cells were incubated for 2 h at RT in the dark with secondary antibodies (Goat Anti-Rabbit IgG H&L, FITC conjugated, Bioss, Beijing, China, bs-0295G-FITC, 1:1000). Finally, nuclei were counterstained with Hoechst 33258 (Thermo, hanghai, China, H3569; 1 μg/mL) for 20 min at RT. Fluorescent images were acquired using a fluorescence microscope (Carl Zeiss, Oberkochen, Germany). Cell count was performed using ImageJ software, and the relative expression of vNP was calculated. The relative expression of vNP protein in the virus group was set to 100%. The relative expression of vNP = (the number of green fluorescent cells in the drug group/the number of blue fluorescent cells in the drug group)/(the number of green fluorescent cells in the virus group/the number of blue fluorescent cells in the virus group) × 100%.

### 4.7. Animal Experiment

A total of 162 specific pathogen-free (SPF) mice aged 6–8 weeks (female, BALB/c, weight 15–20 g) selected by Liaoning Changsheng Biotechnology (Shenyang, China) were randomly assigned to 9 groups (*n* = 18): control group (uninfected group), virus group, positive control group (baloxavir, 5 mg/kg) and drug treatment group. The treatment groups were designated as the NaDC group and the DCA group, each administered orally by gavage at three dose levels: low (25 mg/kg), medium (50 mg/kg), and high (100 mg/kg). Respectively, these were designated as the NaDC-L, NaDC-M, NaDC-H, DCA-L, DCA-M, and DCA-H groups. Within each group, mice were further allocated to a survival cohort (*n* = 12) and a dissection cohort (*n* = 6).

Following a one-week acclimation period, the mice were weighed and then briefly anesthetized with inhaled isoflurane. All mice except those in the control group were intranasally inoculated with 50 μL of H1N1-UI182 virus suspension (in PBS) at a dose of 10 × 50% minimum lethal dose (MLD_50_). At 12 hpi, drug administration was initiated and continued for 5 consecutive days, once daily. The control group was maintained under standard conditions without any treatment. The virus group received 100 μL of PBS via oral gavage. The NaDC and DCA treatment groups were administered their respective drugs at the specified concentrations (100 μL, oral gavage). The baloxavir group received the drug via subcutaneous injection (100 μL). During the 15 days of treatment, mice were weighed daily at a fixed time, and clinical signs, body weight changes, and mortality were recorded. Humane endpoints were defined as weight loss of 25% of initial body weight according to institutional animal welfare guidelines. To analyze survival results, mice whose body weight dropped to meet this condition were euthanized and recorded as dead. Based on these records, survival curves and body weight change curves were generated. On 3 days and 5 dpi, three mice were randomly euthanized from each dissection cohort for tissue harvesting. Subsequently, photographs of the excised lung tissues were taken.

Following excision, the lungs from each mouse were weighed and systematically processed. The trachea and left superior lobe were allocated for viral titration; the left inferior lobe was designated for ELISA; the right anterior lobe was fixed and reserved for H&E staining and immunohistochemical analysis; the right middle lobe was used for RT-qPCR; the right posterior lobe was allocated for WB analysis; the accessory lobe was snap-frozen and stored as a backup sample. All tissue samples were subsequently snap-frozen and stored at −80 °C. The survival rate was calculated as follows: (number of survivors/total number of mice) × 100%. The lung index was calculated as: (lung wet weight/corresponding body weight at harvest) × 100%. The body weight change was expressed as the percentage of initial body weight: (daily body weight/initial body weight) × 100%.

### 4.8. Virus Titer Test

The selected lung tissues were homogenized in Opti-MEM (31985-070, Gibco) medium. The resulting homogenate was subjected to a 10-fold serial dilution series to generate ten different dilutions. Each dilution was inoculated into 9-day-old SPF chicken embryos, which were then incubated at 37 °C for 48 h. After incubation, 50 μL of allantoic fluid was harvested from each egg and transferred to individual wells of a 96-well V-bottom plate. An equal volume (50 μL) of 1% chicken red blood cells (RBC) suspension was then added to each well. The plate was gently mixed and incubated at room temperature (20–25 °C) for 15 min. The HA assay titer is defined as the reciprocal of the highest dilution of the sample that exhibits complete hemagglutination, indicated by a diffuse layer of red blood cells covering the bottom of the well.

### 4.9. Pathological Analysis

Fixed lung tissues were processed routinely: they were dehydrated through a graded ethanol series, cleared in xylene, infiltrated with paraffin wax, and finally embedded in paraffin blocks. Tissue sections of 4–5 μm thickness were cut from the paraffin blocks using a microtome. Subsequently, the sections were deparaffinized, rehydrated, and stained with H&E following a standard staining protocol. The stained sections were scanned using a digital slide scanner (Olympus, Tokyo, Japan) and the images were saved.

### 4.10. ELISA

Frozen lung tissues were retrieved from −80 °C storage and homogenized in ice-cold PBS using a tissue grinder. The homogenate was centrifuged at 14,500× *g* for 15 min at 4 °C. The resulting supernatant was carefully collected for analysis. The concentrations of specific cytokines in the supernatant were quantified using commercial ELISA kits (Elabscience, Wuhan, China) strictly according to the manufacturer’s protocols. Absorbance was measured at the specified wavelength using a microplate reader. Cytokine concentrations were interpolated from a standard curve generated using the recombinant protein standards provided with each kit.

Use the following ELISA kits: TNF-α (Elabscience, E-HSEL-M0009), IFN-β (Elabscience, E-EL-M0033), IL-6 (Elabscience, E-MSEL-M0001), IFN-γ (Elabscience, E-HSEL-M0007), IL-12 (Elabscience, E-MSEL-M0004), IL-1β (Elabscience, E-MSEL-M0003), MCP-1 (Elabscience, E-MSEL-M0012), IP-10/CXCL10 (Elabscience, E-EL-M0021).

### 4.11. RNA Isolation and Quantitative RT-qPCR

To isolate total RNA from mouse lung tissue, we employed the HiPure Universal RNA Extraction Kit (R4130-03, Magen, Guangzhou, China). RNA concentration and purity were assessed using a NanoPhotometer NP80 (Implen, München, Germany). Subsequently, total RNA was reverse-transcribed into complementary DNA (cDNA) using the PrimeScript™ RT Reagent Kit (Takara, RR047A, Kusatsu, Shiga, Japan) according to the manufacturer’s protocol. Finally, gene expression was quantified via RT-qPCR on a Bio-Rad CFX system (Hercules, CA, USA), with *β-actin* mRNA serving as the endogenous control. All primer sequences are provided in Table 2.

### 4.12. In Vivo Experimental Design

Sample Size Planning: Based on conventional practices in influenza antiviral research [55,57,58] and the 3R (Reduction, Optimization, Replacement) ethical principles for animal experiments, 18 mice were included per group to ensure adequate statistical power. Among these, 12 mice were used for continuous survival monitoring, and the remaining 6 were allocated to two different time points for pathological dissection and sampling. This sample size allocation strategy balances animal welfare considerations with the reliability of experimental outcomes, avoiding unnecessary resource consumption.

Randomization: Mice were fully randomized using a random number table generated by an independent third party. Allocation concealment was implemented using sealed envelopes that were opened only at the time of the first drug administration. During the experiment, the cages were arranged in a random block design to avoid potential interference from differences in spatial position, ensure baseline balance between groups, and reduce systematic errors. The indoor relative humidity is maintained at 40%~50%, the indoor temperature is stabilized at 20 °C~25 °C, and the lighting conditions are changed every 12 h to simulate day and night changes.

Blinding Procedures: A fully blinded protocol was implemented throughout the experiment to eliminate observer bias. Drugs and reagents were coded and labeled anonymously. Animal sampling after viral challenge, physiological index measurements, and histopathological evaluation of lung tissues were all performed by blinded personnel unaware of group assignments. Unblinding and statistical analysis were conducted only after all experimental data had been collected.

### 4.13. Statistical Analysis

All statistical analyses were performed using GraphPad Prism 8.0.2 (GraphPad Software, San Diego, CA, USA). Data are presented as mean ± standard deviation (SD). For survival analysis (Figure 2B), the log-rank test was used. For cell viability (Figure 1A–D), virus inhibition (Figure 1E), lung index, viral titer, WB (Figure 1H,I, Figure 2F,G and Figure S2B,C), ELISA, and RT-qPCR, one-way analysis of variance (ANOVA) was used, followed by Dunnett’s post hoc test for comparisons against the control or virus group where applicable. For WB analysis involving multiple treatment groups (Figure 3B), two-way ANOVA was used. Prior to ANOVA, normality was assessed using the Shapiro–Wilk test, and homogeneity of variances was verified using Levene’s test. A *p*-value of less than 0.05 was considered statistically significant, with specific thresholds denoted as follows: * *p* < 0.05, ** *p* < 0.01, *** *p* < 0.001.

## 5. Conclusions

In this study, using a machine learning-assisted virtual screening strategy, we identified two bile acid derivatives, NaDC and DCA, as possessing anti-influenza virus activity. Both compounds demonstrated dose-dependent antiviral activity against the H1N1-UI182 strain in vitro with low cytotoxicity, and improved survival, reduced pulmonary viral load, and alleviated lung injury in a lethal mouse infection model. These phenotypic effects were associated with reduced NF-κB activation and a shift in the balance of pro- and anti-inflammatory cytokines. Notably, should their host-targeting mechanism (such as the calmodulin hypothesis proposed in this study) be confirmed, NaDC and DCA may offer advantages in a higher barrier to drug resistance. Future medicinal chemistry optimization and mechanistic validation will be essential to further develop these compounds toward therapeutic application. Collectively, this study provides a foundation for the continued investigation of NaDC and DCA as anti-influenza leads.

## Figures and Tables

**Table 1:** Preliminary screening results.

Rank	CAS Number	Predicted Binding	Drug Name	Accessibility	Solubility
1	302-95-4	0.5198108	Sodium Deoxycholate	√	√
2	83-44-3	0.5020442	Deoxycholic acid	√	√
3	474-58-8	0.49953148	Daucosterol	√	×
4	516-50-7	0.48015743	Taurodeoxycholic acid	√	√
5	80-99-9	0.47506356	Lathosterol	√	×
6	72962-43-7	0.46969268	Brassinolide	√	√
7	78821-43-9	0.46969268	Epibrassinolide	√	×
8	546-18-9	0.46530408	5*β*-Cholanic acid	√	√
9	22149-69-5	0.46157014	(5*α*)-Stigmastane-3,6-dione	×	×
10	474-62-4	0.46005788	Campesterol	×	×
11	80-97-7	0.45580506	5*α*-Cholestan-3β-ol	×	×

√ represents that the condition is met, while × represents that the condition is not met.

**Table 2:** Primer sequences.

Gene	Primer Sequence (5′ to 3′)
*β-actin*	F: 5′-TGGAATCCCTGTGGGACCATGAAAC-3′ R: 5′-ATCATACTTGGCAGGTTTCTCCAGG-3′
*IFN-α*	F: 5′-GCACCCTGCCTCAGACTCAC-3′ R: 5′-TGCCTGGTCATCTCATGGAAG-3′
*IFN-γ*	F: 5′-AGCCAAATCGTCTCCTTCTACTTC-3′ R: 5′-TGCACCTTGTTGCTGCTGTT-3′
*TNF-α*	F: 5′-AGCCCTGGTATGAACCCATC-3′ R: 5′-GGAATCGGCAAAGTCAAGGT-3′
*IL-1β*	F:5′-TCATCGTGGCAGTGGAAAAG-3′ R: 5′-GGGAAGCAAGGGTCTCAGGT-3′
*IL-6*	F: 5′-AGTTGCCTTCTTGGGACTGATG-3′ R: 5′-GGGAGTGGTATCCTCTGTGAAGTCT-3′
*CCL5*	F: 5′-CTCCTTGCTGCTTTGCCTAC-3′ R: 5′-ACACACCTGGCGGTTCTTTC-3′

## Data Availability

The original contributions presented in this study are included in the article/Supplementary Materials. Further inquiries can be directed to the corresponding author.

## Supplementary Materials

The following supporting information can be downloaded at: https://www.mdpi.com/article/10.3390/ijms27125399/s1.

## Abbreviations

The following abbreviations are used in this manuscript:

IAV	influenza A virus
TCM	traditional Chinese medicine
NaDC	sodium deoxycholate
DCA	deoxycholic acid
NF-κB	nuclear factor kappa-B
IL-10	interleukin-10
WHO	World Health Organization
CDC	Centers for Disease Control and Prevention
GBD	Global Burden of Disease
ARDS	acute respiratory distress syndrome
HA	hemagglutinin
NA	neuraminidase
CaMK	calmodulin-dependent kinase
vNP	viral nucleoprotein
IFN-α/β	interferons (alpha/beta)
CS	cytokine storm
W-7	W-7 hydrochloride
H1N1-UI182	A/Vinig/01/2009 (H1N1)
MDCK	Madin-Darby canine kidney
IL-6	interleukin-6
IL-1β	interleukin-1β
TNF-α	tumor necrosis factor-α
CCK-8	cell counting Kit-8
CC_50_	half-maximal cytotoxic concentration
SAR	structure–activity relationship
STR	structure–toxicity relationship
IFA	immunofluorescence
WB	Western blot
MOI	multiplicity of infection
A549	Human Non-Small Cell Lung Cancer
Vero-E6	African Green Monkey Kidney
hpi	hours post-infection
dpi	days post-infection
HA	Hemagglutination
H&E	Hematoxylin and eosin
IFN-γ	interferon-γ
ELISA	Enzyme-linked immunosorbent assay
RT-qPCR	real-time quantitative PCR
IP-10	interferon gamma-induced protein 10
MCP-1	monocyte chemoattractant protein-1
CCL5	C-C motif chemokine ligand 5
IL-12	interleukin-12
PPI	protein–protein interaction
GO	Gene Ontology
NK	natural killer
SPR	surface plasmon resonance
ITC	isothermal titration calorimetry
DTI	drug–target interaction
Kd	dissociation constant
LGA	Lamarckian genetic algorithm
TCID_50_	50% tissue culture infectious dose
FBS	fetal bovine serum
PBS	phosphate-buffered saline
BSA	bovine serum albumin
RT	room temperature
HRP	horseradish peroxidase
PFA	paraformaldehyde
SPF	specific pathogen-free
MLD_50_	50% minimum lethal dose
RBC	red blood cells
cDNA	complementary DNA
3R	Reduction, Optimization, Replacement
SD	standard deviation
ANOVA	analysis of variance
