# Supplementary material for: Machine Learning and Experimental Verification Identify Anti-Influenza Natural Products
Source: Int J Mol Sci. 2026 Jun 15;27(12):5399. doi: 10.3390/ijms27125399 (PMC13300532; doi:10.3390/ijms27125399)
Supplement: Supplementary file 1 [file ijms-27-05399-s001.zip › Supplement.pdf]

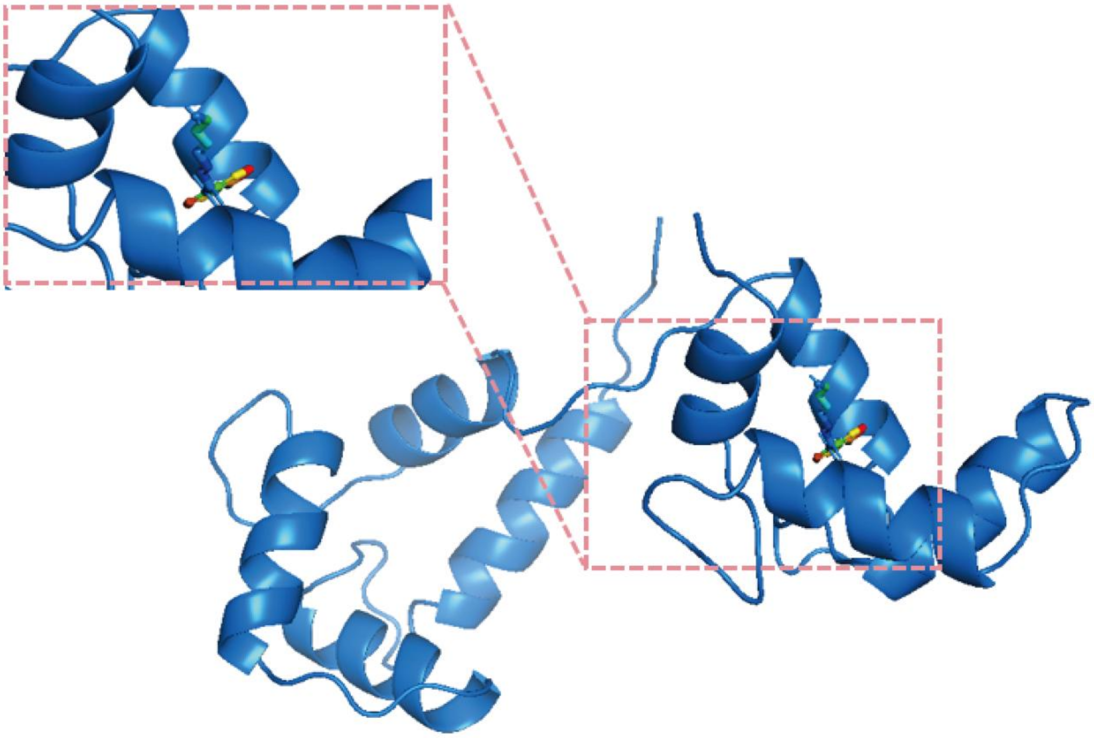

Figure S1. Molecular docking of w-7 with calmodulin.

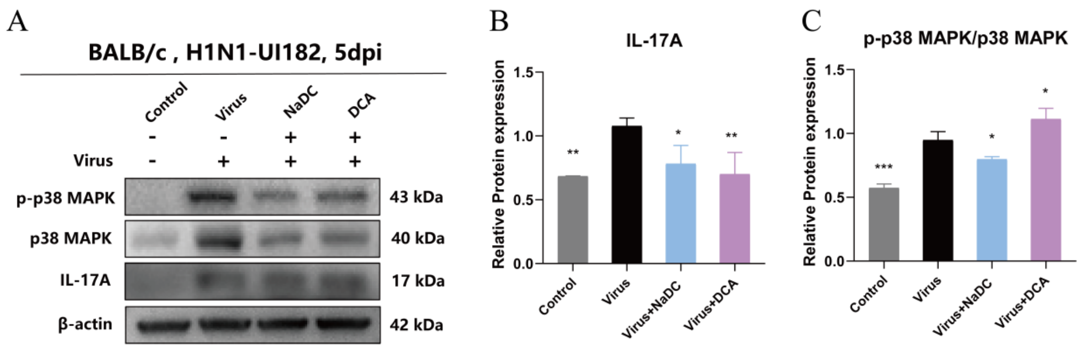

Figure S2. Experimental validation of predicted signaling pathways. (**A**) WB analysis of IL-17A expression and p38 MAPK phosphorylation (p-p38/p38) in lung tissues from H1N1-infected mice treated with NaDC or DCA (100 mg/kg), compared to the virus group. β-actin was used as the loading control for IL-17A; total p38 was used as the loading control for p-p38. (**B**) Quantification of IL-17A protein levels relative to β-actin. (**C**) Quantification of p-p38/p38 ratios. $n = 3$; *$p < 0.05$, **$p < 0.01$, ***$p < 0.001$.

Table S2. $TCID_{50}$ determination of H1N1-UI182 strain

Dilution	CPE positive wells	CPE negative wells	Cumulative positive	Cumulative negative	Cumulative % positive
10^{-1}	5	0	14	0	100%

10^{-2}	5	0	99	0	100%
10^{-3}	4	1	4	1	80%
10^{-4}	0	5	0	6	0
10^{-5}	0	5	0	11	0
10^{-6}	0	5	0	16	0
10^{-7}	0	5	0	21	0

The $TCID_{50}$ value of the H1N1-UI182 virus strain was calculated using the Reed-Muench method and determined to be $10^{4.375}$ $TCID_{50}$/mL.
